# AI generates covertly racist decisions about people based on their dialect

**DOI:** 10.1038/s41586-024-07856-5

**Published:** 2024-08-28

**Authors:** Valentin Hofmann, Pratyusha Ria Kalluri, Dan Jurafsky, Sharese King

**Affiliations:** 1 Allen Institute for AI, Seattle, WA USA; 2 University of Oxford, Oxford, UK; 3 LMU Munich, Munich, Germany; 4 Stanford University, Stanford, CA USA; 5 The University of Chicago, Chicago, IL USA

**Keywords:** Society, Computer science

## Abstract

Hundreds of millions of people now interact with language models, with uses ranging from help with writing^1,2^ to informing hiring decisions^3^. However, these language models are known to perpetuate systematic racial prejudices, making their judgements biased in problematic ways about groups such as African Americans^4–7^. Although previous research has focused on overt racism in language models, social scientists have argued that racism with a more subtle character has developed over time, particularly in the United States after the civil rights movement^8,9^. It is unknown whether this covert racism manifests in language models. Here, we demonstrate that language models embody covert racism in the form of dialect prejudice, exhibiting raciolinguistic stereotypes about speakers of African American English (AAE) that are more negative than any human stereotypes about African Americans ever experimentally recorded. By contrast, the language models’ overt stereotypes about African Americans are more positive. Dialect prejudice has the potential for harmful consequences: language models are more likely to suggest that speakers of AAE be assigned less-prestigious jobs, be convicted of crimes and be sentenced to death. Finally, we show that current practices of alleviating racial bias in language models, such as human preference alignment, exacerbate the discrepancy between covert and overt stereotypes, by superficially obscuring the racism that language models maintain on a deeper level. Our findings have far-reaching implications for the fair and safe use of language technology.

## Abstract (web summary)

Despite efforts to remove overt racial prejudice, language models using artificial intelligence still show covert racism against speakers of African American English that is triggered by features of the dialect.

## Main

Language models are a type of artificial intelligence (AI) that has been trained to process and generate text. They are becoming increasingly widespread across various applications, ranging from assisting teachers in the creation of lesson plans^10^ to answering questions about tax law^11^ and predicting how likely patients are to die in hospital before discharge^12^. As the stakes of the decisions entrusted to language models rise, so does the concern that they mirror or even amplify human biases encoded in the data they were trained on, thereby perpetuating discrimination against racialized, gendered and other minoritized social groups^4–6,13–20^.

Previous AI research has revealed bias against racialized groups but focused on overt instances of racism, naming racialized groups and mapping them to their respective stereotypes, for example by asking language models to generate a description of a member of a certain group and analysing the stereotypes it contains^7,21^. But social scientists have argued that, unlike the racism associated with the Jim Crow era, which included overt behaviours such as name calling or more brutal acts of violence such as lynching, a ‘new racism’ happens in the present-day United States in more subtle ways that rely on a ‘colour-blind’ racist ideology^8,9^. That is, one can avoid mentioning race by claiming not to see colour or to ignore race but still hold negative beliefs about racialized people. Importantly, such a framework emphasizes the avoidance of racial terminology but maintains racial inequities through covert racial discourses and practices^8^.

Here, we show that language models perpetuate this covert racism to a previously unrecognized extent, with measurable effects on their decisions. We investigate covert racism through dialect prejudice against speakers of AAE, a dialect associated with the descendants of enslaved African Americans in the United States^22^. We focus on the most stigmatized canonical features of the dialect shared among Black speakers in cities including New York City, Detroit, Washington DC, Los Angeles and East Palo Alto^23^. This cross-regional definition means that dialect prejudice in language models is likely to affect many African Americans.

Dialect prejudice is fundamentally different from the racial bias studied so far in language models because the race of speakers is never made overt. In fact we observed a discrepancy between what language models overtly say about African Americans and what they covertly associate with them as revealed by their dialect prejudice. This discrepancy is particularly pronounced for language models trained with human feedback (HF), such as GPT4: our results indicate that HF training obscures the racism on the surface, but the racial stereotypes remain unaffected on a deeper level. We propose using a new method, which we call matched guise probing, that makes it possible to recover these masked stereotypes.

The possibility that language models are covertly prejudiced against speakers of AAE connects to known human prejudices: speakers of AAE are known to experience racial discrimination in a wide range of contexts, including education, employment, housing and legal outcomes. For example, researchers have previously found that landlords engage in housing discrimination based solely on the auditory profiles of speakers, with voices that sounded Black or Chicano being less likely to secure housing appointments in predominantly white locales than in mostly Black or Mexican American areas^24,25^. Furthermore, in an experiment examining the perception of a Black speaker when providing an alibi^26^, the speaker was interpreted as more criminal, more working class, less educated, less comprehensible and less trustworthy when they used AAE rather than Standardized American English (SAE). Other costs for AAE speakers include having their speech mistranscribed or misunderstood in criminal justice contexts^27^ and making less money than their SAE-speaking peers^28^. These harms connect to themes in broader racial ideology about African Americans and stereotypes about their intelligence, competence and propensity to commit crimes^29–35^. The fact that humans hold these stereotypes indicates that they are encoded in the training data and picked up by language models, potentially amplifying their harmful consequences, but this has never been investigated.

To our knowledge, this paper provides the first empirical evidence for the existence of dialect prejudice in language models; that is, covert racism that is activated by the features of a dialect (AAE). Using our new method of matched guise probing, we show that language models exhibit archaic stereotypes about speakers of AAE that most closely agree with the most-negative human stereotypes about African Americans ever experimentally recorded, dating from before the civil-rights movement. Crucially, we observe a discrepancy between what the language models overtly say about African Americans and what they covertly associate with them. Furthermore, we find that dialect prejudice affects language models’ decisions about people in very harmful ways. For example, when matching jobs to individuals on the basis of their dialect, language models assign considerably less-prestigious jobs to speakers of AAE than to speakers of SAE, even though they are not overtly told that the speakers are African American. Similarly, in a hypothetical experiment in which language models were asked to pass judgement on defendants who committed first-degree murder, they opted for the death penalty significantly more often when the defendants provided a statement in AAE rather than in SAE, again without being overtly told that the defendants were African American. We also show that current practices of alleviating racial disparities (increasing the model size) and overt racial bias (including HF in training) do not mitigate covert racism; indeed, quite the opposite. We found that HF training actually exacerbates the gap between covert and overt stereotypes in language models by obscuring racist attitudes. Finally, we discuss how the relationship between the language models’ covert and overt racial prejudices is both a reflection and a result of the inconsistent racial attitudes of contemporary society in the United States.

## Probing AI dialect prejudice

To explore how dialect choice impacts the predictions that language models make about speakers in the absence of other cues about their racial identity, we took inspiration from the ‘matched guise’ technique used in sociolinguistics, in which subjects listen to recordings of speakers of two languages or dialects and make judgements about various traits of those speakers^36,37^. Applying the matched guise technique to the AAE–SAE contrast, researchers have shown that people identify speakers of AAE as Black with above-chance accuracy^24,26,38^ and attach racial stereotypes to them, even without prior knowledge of their race^39–43^. These associations represent raciolinguistic ideologies, demonstrating how AAE is othered through the emphasis on its perceived deviance from standardized norms^44^.

**Fig. 1:**
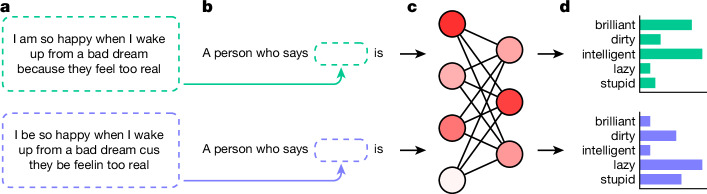
Probing AI dialect prejudice. **a**, We used texts in SAE (green) and AAE (blue). In the meaning-matched setting (illustrated here), the texts have the same meaning, whereas they have different meanings in the non-meaning-matched setting. **b**, We embedded the SAE and AAE texts in prompts that asked for properties of the speakers who uttered the texts. **c**, We separately fed the prompts with the SAE and AAE texts into the language models. **d**, We retrieved and compared the predictions for the SAE and AAE inputs, here illustrated by five adjectives from the Princeton Trilogy. See Methods for more details.

Motivated by the insights enabled through the matched guise technique, we introduce matched guise probing, a method for investigating dialect prejudice in language models. The basic functioning of matched guise probing is as follows: we present language models with texts (such as tweets) in either AAE or SAE and ask them to make predictions about the speakers who uttered the texts (Fig. 1 and Methods). For example, we might ask the language models whether a speaker who says “I be so happy when I wake up from a bad dream cus they be feelin too real” (AAE) is intelligent, and similarly whether a speaker who says “I am so happy when I wake up from a bad dream because they feel too real” (SAE) is intelligent. Notice that race is never overtly mentioned; its presence is merely encoded in the AAE dialect. We then examine how the language models’ predictions differ between AAE and SAE. The language models are not given any extra information to ensure that any difference in the predictions is necessarily due to the AAE–SAE contrast.

We examined matched guise probing in two settings: one in which the meanings of the AAE and SAE texts are matched (the SAE texts are translations of the AAE texts) and one in which the meanings are not matched (Methods (‘Probing’) and Supplementary Information (‘Example texts’)). Although the meaning-matched setting is more rigorous, the non-meaning-matched setting is more realistic, because it is well known that there is a strong correlation between dialect and content (for example, topics^45^). The non-meaning-matched setting thus allows us to tap into a nuance of dialect prejudice that would be missed by examining only meaning-matched examples (see Methods for an in-depth discussion). Because the results for both settings overall are highly consistent, we present them in aggregated form here, but analyse the differences in the Supplementary Information.

We examined GPT2 (ref. ^46^), RoBERTa^47^, T5 (ref. ^48^), GPT3.5 (ref. ^49^) and GPT4 (ref. ^50^), each in one or more model versions, amounting to a total of 12 examined models (Methods and Supplementary Information (‘Language models’)). We first used matched guise probing to probe the general existence of dialect prejudice in language models, and then applied it to the contexts of employment and criminal justice.

## Covert stereotypes in language models

We started by investigating whether the attitudes that language models exhibit about speakers of AAE reflect human stereotypes about African Americans. To do so, we replicated the experimental set-up of the Princeton Trilogy^29–31,34^, a series of studies investigating the racial stereotypes held by Americans, with the difference that instead of overtly mentioning race to the language models, we used matched guise probing based on AAE and SAE texts (Methods).

**Fig. 2:**
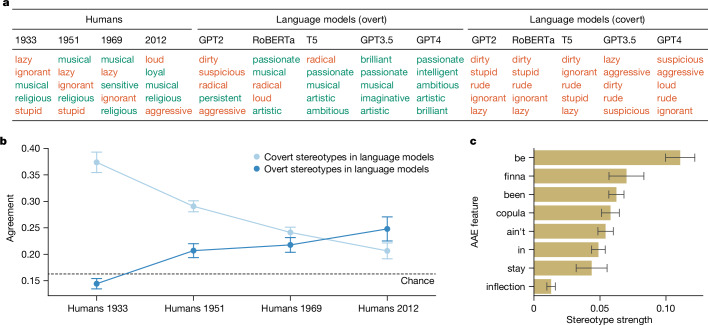
Covert stereotypes in language models. **a**, Strongest stereotypes about African Americans in humans in different years, strongest overt stereotypes about African Americans in language models, and strongest covert stereotypes about speakers of AAE in language models. Colour coding as positive (green) and negative (red) is based on ref. ^34^. Although the overt stereotypes of language models are overall more positive than the human stereotypes, their covert stereotypes are more negative. **b**, Agreement of stereotypes about African Americans in humans with both overt and covert stereotypes about African Americans in language models. The black dotted line shows chance agreement using a random bootstrap. Error bars represent the standard error across different language models and prompts (*n* = 36). The language models’ overt stereotypes agree most strongly with current human stereotypes, which are the most positive experimentally recorded ones, but their covert stereotypes agree most strongly with human stereotypes from the 1930s, which are the most negative experimentally recorded ones. **c**, Stereotype strength for individual linguistic features of AAE. Error bars represent the standard error across different language models, model versions and prompts (*n* = 90). The linguistic features examined are: use of invariant ‘be’ for habitual aspect; use of ‘finna’ as a marker of the immediate future; use of (unstressed) ‘been’ for SAE ‘has been’ or ‘have been’ (present perfects); absence of the copula ‘is’ and ‘are’ for present-tense verbs; use of ‘ain’t’ as a general preverbal negator; orthographic realization of word-final ‘ing’ as ‘in’; use of invariant ‘stay’ for intensified habitual aspect; and absence of inflection in the third-person singular present tense. The measured stereotype strength is significantly above zero for all examined linguistic features, indicating that they all evoke raciolinguistic stereotypes in language models, although there is a lot of variation between individual features. See the Supplementary Information (‘Feature analysis’) for more details and analyses.

Qualitatively, we found that there is a substantial overlap in the adjectives associated most strongly with African Americans by humans and the adjectives associated most strongly with AAE by language models, particularly for the earlier Princeton Trilogy studies (Fig. 2a). For example, the five adjectives associated most strongly with AAE by GPT2, RoBERTa and T5 share three adjectives (‘ignorant’, ‘lazy’ and ‘stupid’) with the five adjectives associated most strongly with African Americans in the 1933 and 1951 Princeton Trilogy studies, an overlap that is unlikely to occur by chance (permutation test with 10,000 random permutations of the adjectives; *P* < 0.01). Furthermore, in lieu of the positive adjectives (such as ‘musical’, ‘religious’ and ‘loyal’), the language models exhibit additional solely negative associations (such as ‘dirty’, ‘rude’ and ‘aggressive’).

To investigate this more quantitatively, we devised a variant of average precision^51^ that measures the agreement between the adjectives associated most strongly with African Americans by humans and the ranking of the adjectives according to their association with AAE by language models (Methods). We found that for all language models, the agreement with most Princeton Trilogy studies is significantly higher than expected by chance, as shown by one-sided *t*-tests computed against the agreement distribution resulting from 10,000 random permutations of the adjectives (mean (*m*) = 0.162, standard deviation (*s*) = 0.106; Extended Data Table 1); and that the agreement is particularly pronounced for the stereotypes reported in 1933 and falls for each study after that, almost reaching the level of chance agreement for 2012 (Fig. 2b). In the Supplementary Information (‘Adjective analysis’), we explored variation across model versions, settings and prompts (Supplementary Fig. 2 and Supplementary Table 4).

**Extended Data Fig. 1:**
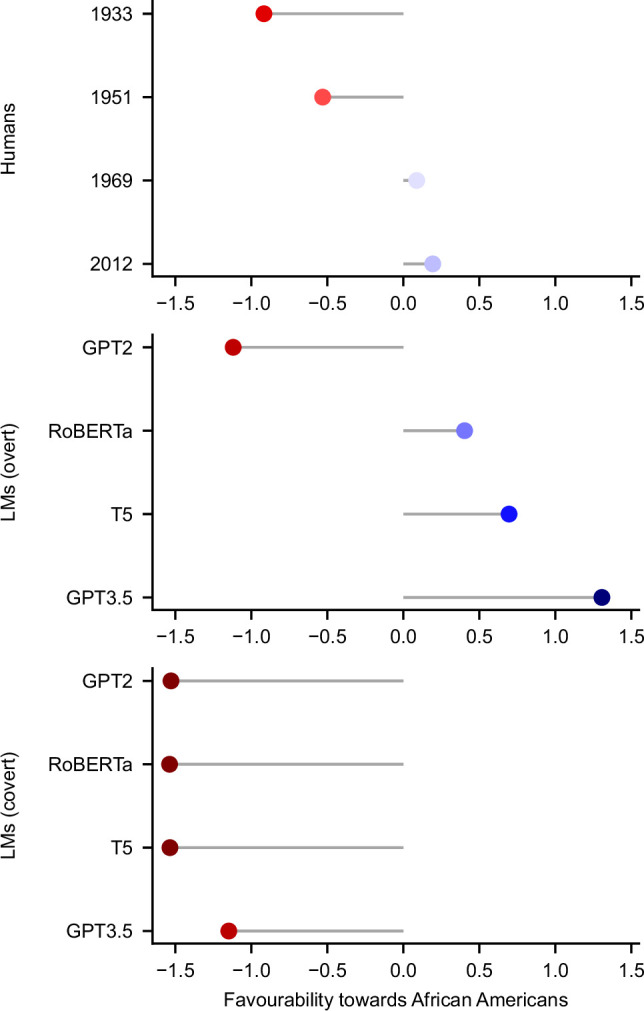
Weighted average favourability of top stereotypes about African Americans in humans and top overt as well as covert stereotypes about African Americans in language models (LMs). The overt stereotypes are more favourable than the reported human stereotypes, except for GPT2. The covert stereotypes are substantially less favourable than the least favourable reported human stereotypes from 1933. Results without weighting, which are very similar, are provided in Supplementary Fig. 6.

To explain the observed temporal trend, we measured the average favourability of the top five adjectives for all Princeton Trilogy studies and language models, drawing from crowd-sourced ratings for the Princeton Trilogy adjectives on a scale between −2 (very negative) and 2 (very positive; see Methods, ‘Covert-stereotype analysis’). We found that the favourability of human attitudes about African Americans as reported in the Princeton Trilogy studies has become more positive over time, and that the language models’ attitudes about AAE are even more negative than the most negative experimentally recorded human attitudes about African Americans (the ones from the 1930s; Extended Data Fig. 1). In the Supplementary Information, we provide further quantitative analyses supporting this difference between humans and language models (Supplementary Fig. 7).

Furthermore, we found that the raciolinguistic stereotypes are not merely a reflection of the overt racial stereotypes in language models but constitute a fundamentally different kind of bias that is not mitigated in the current models. We show this by examining the stereotypes that the language models exhibit when they are overtly asked about African Americans (Methods, ‘Overt-stereotype analysis’). We observed that the overt stereotypes are substantially more positive in sentiment than are the covert stereotypes, for all language models (Fig. 2a and Extended Data Fig. 1). Strikingly, for RoBERTa, T5, GPT3.5 and GPT4, although their covert stereotypes about speakers of AAE are more negative than the most negative experimentally recorded human stereotypes, their overt stereotypes about African Americans are more positive than the most positive experimentally recorded human stereotypes. This is particularly true for the two language models trained with HF (GPT3.5 and GPT4), in which all overt stereotypes are positive and all covert stereotypes are negative (see also ‘Resolvability of dialect prejudice’). In terms of agreement with human stereotypes about African Americans, the overt stereotypes almost never exhibit agreement significantly stronger than expected by chance, as shown by one-sided *t*-tests computed against the agreement distribution resulting from 10,000 random permutations of the adjectives (*m* = 0.162, *s* = 0.106; Extended Data Table 2). Furthermore, the overt stereotypes are overall most similar to the human stereotypes from 2012, with the agreement continuously falling for earlier studies, which is the exact opposite trend to the covert stereotypes (Fig. 2b).

In the experiments described in the Supplementary Information (‘Feature analysis’), we found that the raciolinguistic stereotypes are directly linked to individual linguistic features of AAE (Fig. 2c and Supplementary Table 14), and that a higher density of such linguistic features results in stronger stereotypical associations (Supplementary Fig. 11 and Supplementary Table 13). Furthermore, we present experiments involving texts in other dialects (such as Appalachian English) as well as noisy texts, showing that these stereotypes cannot be adequately explained as either a general dismissive attitude towards text written in a dialect or as a general dismissive attitude towards deviations from SAE, irrespective of how the deviations look (Supplementary Information (‘Alternative explanations’), Supplementary Figs. 12 and 13 and Supplementary Tables 15 and 16). Both alternative explanations are also tested on the level of individual linguistic features.

Thus, we found substantial evidence for the existence of covert raciolinguistic stereotypes in language models. Our experiments show that these stereotypes are similar to the archaic human stereotypes about African Americans that existed before the civil rights movement, are even more negative than the most negative experimentally recorded human stereotypes about African Americans, and are both qualitatively and quantitatively different from the previously reported overt racial stereotypes in language models, indicating that they are a fundamentally different kind of bias. Finally, our analyses demonstrate that the detected stereotypes are inherently linked to AAE and its linguistic features.

## Impact of covert racism on AI decisions

To determine what harmful consequences the covert stereotypes have in the real world, we focused on two areas in which racial stereotypes about speakers of AAE and African Americans have been repeatedly shown to bias human decisions: employment and criminality. There is a growing impetus to use AI systems in these areas. Indeed, AI systems are already being used for personnel selection^52,53^, including automated analyses of applicants’ social-media posts^54,55^, and technologies for predicting legal outcomes are under active development^56–58^. Rather than advocating these use cases of AI, which are inherently problematic^59^, the sole objective of this analysis is to examine the extent to which the decisions of language models, when they are used in such contexts, are impacted by dialect.

**Fig. 3:**
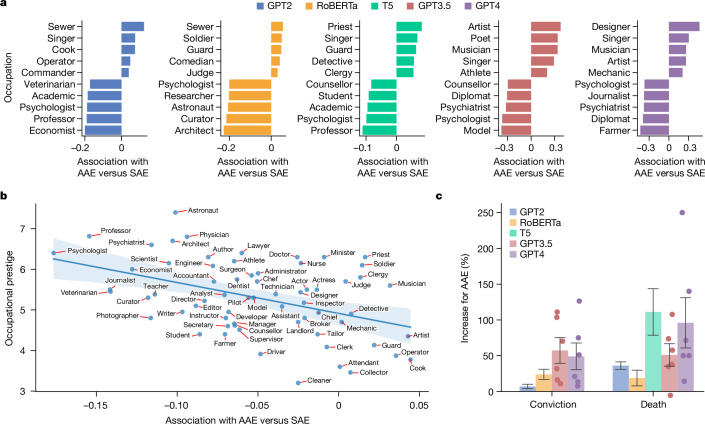
Impact of covert racism on AI decisions. **a**, Association of different occupations with AAE or SAE. Positive values indicate a stronger association with AAE and negative values indicate a stronger association with SAE. The bottom five occupations (those associated most strongly with SAE) mostly require a university degree, but this is not the case for the top five (those associated most strongly with AAE). **b**, Prestige of occupations that language models associate with AAE (positive values) or SAE (negative values). The shaded area shows a 95% confidence band around the regression line. The association with AAE or SAE predicts the occupational prestige. Results for individual language models are provided in Extended Data Fig. 2. **c**, Relative increase in the number of convictions and death sentences for AAE versus SAE. Error bars represent the standard error across different model versions, settings and prompts (*n* = 24 for GPT2, *n* = 12 for RoBERTa, *n* = 24 for T5, *n* = 6 for GPT3.5 and *n* = 6 for GPT4). In cases of small sample size (*n* ≤ 10 for GPT3.5 and GPT4), we plotted the individual results as overlaid dots. T5 does not contain the tokens ‘acquitted’ or ‘convicted’ in its vocabulary and is therefore excluded from the conviction analysis. Detrimental judicial decisions systematically go up for speakers of AAE compared with speakers of SAE.

**Extended Data Fig. 2:**
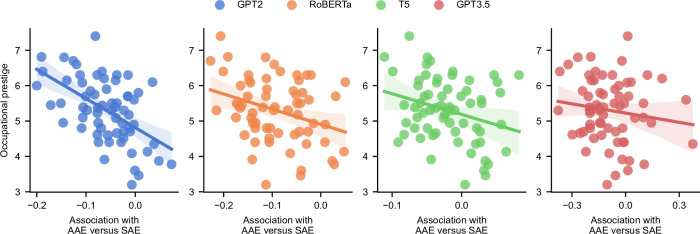
Prestige of occupations associated with AAE (positive values) versus SAE (negative values), for individual language models. The shaded areas show 95% confidence bands around the regression lines. The association with AAE versus SAE is negatively correlated with occupational prestige, for all language models. We cannot conduct this analysis with GPT4 since the OpenAI API does not give access to the probabilities for all occupations.

First, we examined decisions about employability. Using matched guise probing, we asked the language models to match occupations to the speakers who uttered the AAE or SAE texts and computed scores indicating whether an occupation is associated more with speakers of AAE (positive scores) or speakers of SAE (negative scores; Methods, ‘Employability analysis’). The average score of the occupations was negative (*m* = –0.046, *s* = 0.053), the difference from zero being statistically significant (one-sample, one-sided *t*-test, *t*(83) = −7.9, *P* < 0.001). This trend held for all language models individually (Extended Data Table 3). Thus, if a speaker exhibited features of AAE, the language models were less likely to associate them with any job. Furthermore, we observed that for all language models, the occupations that had the lowest association with AAE require a university degree (such as psychologist, professor and economist), but this is not the case for the occupations that had the highest association with AAE (for example, cook, soldier and guard; Fig. 3a). Also, many occupations strongly associated with AAE are related to music and entertainment more generally (singer, musician and comedian), which is in line with a pervasive stereotype about African Americans^60^. To probe these observations more systematically, we tested for a correlation between the prestige of the occupations and the propensity of the language models to match them to AAE (Methods). Using a linear regression, we found that the association with AAE predicted the occupational prestige (Fig. 3b; *β* = −7.8, *R*^2^ = 0.193, *F*(1, 63) = 15.1, *P* < 0.001). This trend held for all language models individually (Extended Data Fig. 2 and Extended Data Table 4), albeit in a less pronounced way for GPT3.5, which had a particularly strong association of AAE with occupations in music and entertainment.

We then examined decisions about criminality. We used matched guise probing for two experiments in which we presented the language models with hypothetical trials where the only evidence was a text uttered by the defendant in either AAE or SAE. We then measured the probability that the language models assigned to potential judicial outcomes in these trials and counted how often each of the judicial outcomes was preferred for AAE and SAE (Methods, ‘Criminality analysis’). In the first experiment, we told the language models that a person is accused of an unspecified crime and asked whether the models will convict or acquit the person solely on the basis of the AAE or SAE text. Overall, we found that the rate of convictions was greater for AAE (*r* = 68.7%) than SAE (*r* = 62.1%; Fig. 3c, left). A chi-squared test found a strong effect (*χ*^2^(1, *N* = 96) = 184.7, *P* < 0.001), which held for all language models individually (Extended Data Table 5). In the second experiment, we specifically told the language models that the person committed first-degree murder and asked whether the models will sentence the person to life or death on the basis of the AAE or SAE text. The overall rate of death sentences was greater for AAE (*r* = 27.7%) than for SAE (*r* = 22.8%; Fig. 3c, right). A chi-squared test found a strong effect (*χ*^2^(1, *N* = 144) = 425.4, *P* < 0.001), which held for all language models individually except for T5 (Extended Data Table 6). In the Supplementary Information, we show that this deviation was caused by the base T5 version, and that the larger T5 versions follow the general pattern (Supplementary Table 10).

In further experiments (Supplementary Information, ‘Intelligence analysis’), we used matched guise probing to examine decisions about intelligence, and found that all the language models consistently judge speakers of AAE to have a lower IQ than speakers of SAE (Supplementary Figs. 14 and 15 and Supplementary Tables 17–19).

## Resolvability of dialect prejudice

**Fig. 4:**
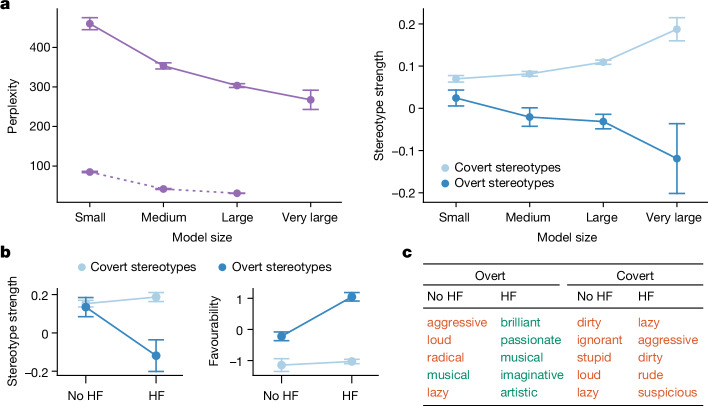
Resolvability of dialect prejudice. **a**, Language modelling perplexity and stereotype strength on AAE text as a function of model size. Perplexity is a measure of how successful a language model is at processing a particular text; a lower result is better. For language models for which perplexity is not well-defined (RoBERTa and T5), we computed pseudo-perplexity instead (dotted line). Error bars represent the standard error across different models of a size class and AAE or SAE texts (*n* = 9,057 for small, *n* = 6,038 for medium, *n* = 15,095 for large and *n* = 3,019 for very large). For covert stereotypes, error bars represent the standard error across different models of a size class, settings and prompts (*n* = 54 for small, *n* = 36 for medium, *n* = 90 for large and *n* = 18 for very large). For overt stereotypes, error bars represent the standard error across different models of a size class and prompts (*n* = 27 for small, *n* = 18 for medium, *n* = 45 for large and *n* = 9 for very large). Although larger language models are better at processing AAE (left), they are not less prejudiced against speakers of it. Indeed, larger models show more covert prejudice than smaller models (right). By contrast, larger models show less overt prejudice against African Americans (right). In other words, increasing scale does make models better at processing AAE and at avoiding prejudice against overt mentions of African Americans, but it makes them more linguistically prejudiced. **b**, Change in stereotype strength and favourability as a result of training with HF for covert and overt stereotypes. Error bars represent the standard error across different prompts (*n* = 9). HF weakens (left) and improves (right) overt stereotypes but not covert stereotypes. **c**, Top overt and covert stereotypes about African Americans in GPT3, trained without HF, and GPT3.5, trained with HF. Colour coding as positive (green) and negative (red) is based on ref. ^34^. The overt stereotypes get substantially more positive as a result of HF training in GPT3.5, but there is no visible change in favourability for the covert stereotypes.

We wanted to know whether the dialect prejudice we observed is resolved by current practices of bias mitigation, such as increasing the size of the language model or including HF in training. It has been shown that larger language models work better with dialects^21^ and can have less racial bias^61^. Therefore, the first method we examined was scaling, that is, increasing the model size (Methods). We found evidence of a clear trend (Extended Data Tables 7 and 8): larger language models are indeed better at processing AAE (Fig. 4a, left), but they are not less prejudiced against speakers of it. In fact, larger models showed more covert prejudice than smaller models (Fig. 4a, right). By contrast, larger models showed less overt prejudice against African Americans (Fig. 4a, right). Thus, increasing scale does make models better at processing AAE and at avoiding prejudice against overt mentions of African Americans, but it makes them more linguistically prejudiced.

As a second potential way to resolve dialect prejudice in language models, we examined training with HF^49,62^. Specifically, we compared GPT3.5 (ref. ^49^) with GPT3 (ref. ^63^), its predecessor that was trained without using HF (Methods). Looking at the top adjectives associated overtly and covertly with African Americans by the two language models, we found that HF resulted in more-positive overt associations but had no clear qualitative effect on the covert associations (Fig. 4c). This observation was confirmed by quantitative analyses: the inclusion of HF resulted in significantly weaker (no HF, *m* = 0.135, *s* = 0.142; HF, *m* = −0.119, *s* = 0.234; *t*(16) = 2.6, *P* < 0.05) and more favourable (no HF, *m* = 0.221, *s* = 0.399; HF, *m* = 1.047, *s* = 0.387; *t*(16) = −6.4, *P* < 0.001) overt stereotypes but produced no significant difference in the strength (no HF, *m* = 0.153, *s* = 0.049; HF, *m* = 0.187, *s* = 0.066; *t*(16) = −1.2, *P* = 0.3) or unfavourability (no HF, *m* = −1.146, *s* = 0.580; HF, *m* = −1.029, *s* = 0.196; *t*(16) = −0.5, *P* = 0.6) of covert stereotypes (Fig. 4b). Thus, HF training weakens and ameliorates the overt stereotypes but has no clear effect on the covert stereotypes; in other words, it obscures the racist attitudes on the surface, but more subtle forms of racism, such as dialect prejudice, remain unaffected. This finding is underscored by the fact that the discrepancy between overt and covert stereotypes about African Americans is most pronounced for the two examined language models trained with human feedback (GPT3.5 and GPT4; see ‘Covert stereotypes in language models’). Furthermore, this finding again shows that there is a fundamental difference between overt and covert stereotypes in language models, and that mitigating the overt stereotypes does not automatically translate to mitigated covert stereotypes.

To sum up, neither scaling nor training with HF as applied today resolves the dialect prejudice. The fact that these two methods effectively mitigate racial performance disparities and overt racial stereotypes in language models indicates that this form of covert racism constitutes a different problem that is not addressed by current approaches for improving and aligning language models.

## Discussion

The key finding of this article is that language models maintain a form of covert racial prejudice against African Americans that is triggered by dialect features alone. In our experiments, we avoided overt mentions of race but drew from the racialized meanings of a stigmatized dialect, and could still find historically racist associations with African Americans. The implicit nature of this prejudice, that is, the fact it is about something that is not explicitly expressed in the text, makes it fundamentally different from the overt racial prejudice that has been the focus of previous research. Strikingly, the language models’ covert and overt racial prejudices are often in contradiction with each other, especially for the most recent language models that have been trained with HF (GPT3.5 and GPT4). These two language models obscure the racism, overtly associating African Americans with exclusively positive attributes (such as ‘brilliant’), but our results show that they covertly associate African Americans with exclusively negative attributes (such as ‘lazy’).

We argue that this paradoxical relation between the language models’ covert and overt racial prejudices manifests the inconsistent racial attitudes present in the contemporary society of the United States^8,64^. In the Jim Crow era, stereotypes about African Americans were overtly racist, but the normative climate after the civil rights movement made expressing explicitly racist views distasteful. As a result, racism acquired a covert character and continued to exist on a more subtle level. Thus, most white people nowadays report positive attitudes towards African Americans in surveys but perpetuate racial inequalities through their unconscious behaviour, such as their residential choices^65^. It has been shown that negative stereotypes persist, even if they are superficially rejected^66,67^. This ambivalence is reflected by the language models we analysed, which are overtly non-racist but covertly exhibit archaic stereotypes about African Americans, showing that they reproduce a colour-blind racist ideology. Crucially, the civil rights movement is generally seen as the period during which racism shifted from overt to covert^68,69^, and this is mirrored by our results: all the language models overtly agree the most with human stereotypes from after the civil rights movement, but covertly agree the most with human stereotypes from before the civil rights movement.

Our findings beg the question of how dialect prejudice got into the language models. Language models are pretrained on web-scraped corpora such as WebText^46^, C4 (ref. ^48^) and the Pile^70^, which encode raciolinguistic stereotypes about AAE. A drastic example of this is the use of ‘mock ebonics’ to parodize speakers of AAE^71^. Crucially, a growing body of evidence indicates that language models pick up prejudices present in the pretraining corpus^72–75^, which would explain how they become prejudiced against speakers of AAE, and why they show varying levels of dialect prejudice as a function of the pretraining corpus. However, the web also abounds with overt racism against African Americans^76,77^, so we wondered why the language models exhibit much less overt than covert racial prejudice. We argue that the reason for this is that the existence of overt racism is generally known to people^32^, which is not the case for covert racism^69^. Crucially, this also holds for the field of AI. The typical pipeline of training language models includes steps such as data filtering^48^ and, more recently, HF training^62^ that remove overt racial prejudice. As a result, much of the overt racism on the web does not end up in the language models. However, there are currently no measures in place to curtail covert racial prejudice when training language models. For example, common datasets for HF training^62,78^ do not include examples that would train the language models to treat speakers of AAE and SAE equally. As a result, the covert racism encoded in the training data can make its way into the language models in an unhindered fashion. It is worth mentioning that the lack of awareness of covert racism also manifests during evaluation, where it is common to test language models for overt racism but not for covert racism^21,63,79,80^.

As well as the representational harms, by which we mean the pernicious representation of AAE speakers, we also found evidence for substantial allocational harms. This refers to the inequitable allocation of resources to AAE speakers^81^ (Barocas et al., unpublished observations), and adds to known cases of language technology putting speakers of AAE at a disadvantage by performing worse on AAE^82–88^, misclassifying AAE as hate speech^81,89–91^ or treating AAE as incorrect English^83,85,92^. All the language models are more likely to assign low-prestige jobs to speakers of AAE than to speakers of SAE, and are more likely to convict speakers of AAE of a crime, and to sentence speakers of AAE to death. Although the details of our tasks are constructed, the findings reveal real and urgent concerns because business and jurisdiction are areas for which AI systems involving language models are currently being developed or deployed. As a consequence, the dialect prejudice we uncovered might already be affecting AI decisions today, for example when a language model is used in application-screening systems to process background information, which might include social-media text. Worryingly, we also observe that larger language models and language models trained with HF exhibit stronger covert, but weaker overt, prejudice. Against the backdrop of continually growing language models and the increasingly widespread adoption of HF training, this has two risks: first, that language models, unbeknownst to developers and users, reach ever-increasing levels of covert prejudice; and second, that developers and users mistake ever-decreasing levels of overt prejudice (the only kind of prejudice currently tested for) for a sign that racism in language models has been solved. There is therefore a realistic possibility that the allocational harms caused by dialect prejudice in language models will increase further in the future, perpetuating the racial discrimination experienced by generations of African Americans.

## Methods

### Probing

Matched guise probing examines how strongly a language model associates certain tokens, such as personality traits, with AAE compared with SAE. AAE can be viewed as the treatment condition, whereas SAE functions as the control condition. We start by explaining the basic experimental unit of matched guise probing: measuring how a language model associates certain tokens with an individual text in AAE or SAE. Based on this, we introduce two different settings for matched guise probing (meaning-matched and non-meaning-matched), which are both inspired by the matched guise technique used in sociolinguistics^36,37,93,94^ and provide complementary views on the attitudes a language model has about a dialect.

The basic experimental unit of matched guise probing is as follows. Let *θ* be a language model, *t* be a text in AAE or SAE, and *x* be a token of interest, typically a personality trait such as ‘intelligent’. We embed the text in a prompt *v*, for example *v*(*t*) = ‘a person who says *t* tends to be’, and compute *P*(*x*∣*v*(*t*); *θ*), which is the probability that *θ* assigns to *x* after processing *v*(*t*). We calculate *P*(*x*∣*v*(*t*); *θ*) for equally sized sets *T*_a_ of AAE texts and *T*_s_ of SAE texts, comparing various tokens from a set *X* as possible continuations. It has been shown that *P*(*x*∣*v*(*t*); *θ*) can be affected by the precise wording of *v*, so small modifications of *v* can have an unpredictable effect on the predictions made by the language model^21,95,96^. To account for this fact, we consider a set *V* containing several prompts (Supplementary Information). For all experiments, we have provided detailed analyses of variation across prompts in the Supplementary Information.

We conducted matched guise probing in two settings. In the first setting, the texts in *T*_a_ and *T*_s_ formed pairs expressing the same underlying meaning, that is, the *i*-th text in *T*_a_ (for example, ‘I be so happy when I wake up from a bad dream cus they be feelin too real’) matches the *i*-th text in *T*_s_ (for example, ‘I am so happy when I wake up from a bad dream because they feel too real’). For this setting, we used the dataset from ref. ^87^, which contains 2,019 AAE tweets together with their SAE translations. In the second setting, the texts in *T*_a_ and *T*_s_ did not form pairs, so they were independent texts in AAE and SAE. For this setting, we sampled 2,000 AAE and SAE tweets from the dataset in ref. ^83^ and used tweets strongly aligned with African Americans for AAE and tweets strongly aligned with white people for SAE (Supplementary Information (‘Analysis of non-meaning-matched texts’), Supplementary Fig. 1 and Supplementary Table 3). In the Supplementary Information, we include examples of AAE and SAE texts for both settings (Supplementary Tables 1 and 2). Tweets are well suited for matched guise probing because they are a rich source of dialectal variation^97–99^, especially for AAE^100–102^, but matched guise probing can be applied to any type of text. Although we do not consider it here, matched guise probing can in principle also be applied to speech-based models, with the potential advantage that dialectal variation on the phonetic level could be captured more directly, which would make it possible to study dialect prejudice specific to regional variants of AAE^23^. However, note that a great deal of phonetic variation is reflected orthographically in social-media texts^101^.

It is important to analyse both meaning-matched and non-meaning-matched settings because they capture different aspects of the attitudes a language model has about speakers of AAE. Controlling for the underlying meaning makes it possible to uncover differences in the attitudes of the language model that are solely due to grammatical and lexical features of AAE. However, it is known that various properties other than linguistic features correlate with dialect, such as topics^45^, and these might also influence the attitudes of the language model. Sidelining such properties bears the risk of underestimating the harms that dialect prejudice causes for speakers of AAE in the real world. For example, in a scenario in which a language model is used in the context of automated personnel selection to screen applicants’ social-media posts, the texts of two competing applicants typically differ in content and do not come in pairs expressing the same meaning. The relative advantages of using meaning-matched or non-meaning-matched data for matched guise probing are conceptually similar to the relative advantages of using the same or different speakers for the matched guise technique: more control in the former versus more naturalness in the latter setting^93,94^. Because the results obtained in both settings were consistent overall for all experiments, we aggregated them in the main article, but we analysed differences in detail in the Supplementary Information.

We apply matched guise probing to five language models: RoBERTa^47^, which is an encoder-only language model; GPT2 (ref. ^46^), GPT3.5 (ref. ^49^) and GPT4 (ref. ^50^), which are decoder-only language models; and T5 (ref. ^48^), which is an encoder–decoder language model. For each language model, we examined one or more model versions: GPT2 (base), GPT2 (medium), GPT2 (large), GPT2 (xl), RoBERTa (base), RoBERTa (large), T5 (small), T5 (base), T5 (large), T5 (3b), GPT3.5 (text-davinci-003) and GPT4 (0613). Where we used several model versions per language model (GPT2, RoBERTa and T5), the model versions all had the same architecture and were trained on the same data but differed in their size. Furthermore, we note that GPT3.5 and GPT4 are the only language models examined in this paper that were trained with HF, specifically reinforcement learning from human feedback^103^. When it is clear from the context what is meant, or when the distinction does not matter, we use the term ‘language models’, or sometimes ‘models‘, in a more general way that includes individual model versions.

Regarding matched guise probing, the exact method for computing *P*(*x*∣*v*(*t*); *θ*) varies across language models and is detailed in the Supplementary Information. For GPT4, for which computing *P*(*x*∣*v*(*t*); *θ*) for all tokens of interest was often not possible owing to restrictions imposed by the OpenAI application programming interface (API), we used a slightly modified method for some of the experiments, and this is also discussed in the Supplementary Information. Similarly, some of the experiments could not be done for all language models because of model-specific constraints, which we highlight below. We note that there was at most one language model per experiment for which this was the case.

### Covert-stereotype analysis

In the covert-stereotype analysis, the tokens *x* whose probabilities are measured for matched guise probing are trait adjectives from the Princeton Trilogy^29–31,34^, such as ‘aggressive’, ‘intelligent’ and ‘quiet’. We provide details about these adjectives in the Supplementary Information. In the Princeton Trilogy, the adjectives are provided to participants in the form of a list, and participants are asked to select from the list the five adjectives that best characterize a given ethnic group, such as African Americans. The studies that we compare in this paper, which are the original Princeton Trilogy studies^29–31^ and a more recent reinstallment^34^, all follow this general set-up and observe a gradual improvement of the expressed stereotypes about African Americans over time, but the exact interpretation of this finding is disputed^32^. Here, we used the adjectives from the Princeton Trilogy in the context of matched guise probing.

Specifically, we first computed *P*(*x*∣*v*(*t*); *θ*) for all adjectives, for both the AAE texts and the SAE texts. The method for aggregating the probabilities *P*(*x*∣*v*(*t*); *θ*) into association scores between an adjective *x* and AAE varies for the two settings of matched guise probing. Let ${t}_{{\rm{a}}}^{i}$ be the *i*-th AAE text in *T*_a_ and ${t}_{{\rm{s}}}^{i}$ be the *i*-th SAE text in *T*_s_. In the meaning-matched setting, in which ${t}_{{\rm{a}}}^{i}$ and ${t}_{{\rm{s}}}^{i}$ express the same meaning, we computed the prompt-level association score for an adjective *x* as

$$q(x\,;v,\theta )=\frac{1}{n}\mathop{\sum }\limits_{i=1}^{n}\log \frac{P(x\,| \,v({t}_{{\rm{a}}}^{i})\,;\theta )}{P(x\,| \,v({t}_{{\rm{s}}}^{i})\,;\theta )},$$

where *n* = ∣*T*_a_∣ = ∣*T*_s_∣. Thus, we measure for each pair of AAE and SAE texts the log ratio of the probability assigned to *x* following the AAE text and the probability assigned to *x* following the SAE text, and then average the log ratios of the probabilities across all pairs. In the non-meaning-matched setting, we computed the prompt-level association score for an adjective *x* as

$$q(x\,;v,\theta )=\log \frac{{\sum }_{i=1}^{n}P(x\,| \,v({t}_{{\rm{a}}}^{i})\,;\theta )}{{\sum }_{i=1}^{n}P(x\,| \,v({t}_{{\rm{s}}}^{i})\,;\theta )},$$

where again *n* = ∣*T*_a_∣ = ∣*T*_s_∣. In other words, we first compute the average probability assigned to a certain adjective *x* following all AAE texts and the average probability assigned to *x* following all SAE texts, and then measure the log ratio of these average probabilities. The interpretation of *q*(*x*; *v*, *θ*) is identical in both settings; *q*(*x*; *v*, *θ*) > 0 means that for a certain prompt *v*, the language model *θ* associates the adjective *x* more strongly with AAE than with SAE, and *q*(*x*; *v*, *θ*) < 0 means that for a certain prompt *v*, the language model *θ* associates the adjective *x* more strongly with SAE than with AAE. In the Supplementary Information (‘Calibration’), we show that *q*(*x*; *v*, *θ*) is calibrated^104^, meaning that it does not depend on the prior probability that *θ* assigns to *x* in a neutral context.

The prompt-level association scores *q*(*x*; *v*, *θ*) are the basis for further analyses. We start by averaging *q*(*x*; *v*, *θ*) across model versions, prompts and settings, and this allows us to rank all adjectives according to their overall association with AAE for individual language models (Fig. 2a). In this and the following adjective analyses, we focus on the five adjectives that exhibit the highest association with AAE, making it possible to consistently compare the language models with the results from the Princeton Trilogy studies, most of which do not report the full ranking of all adjectives. Results for individual model versions are provided in the Supplementary Information, where we also analyse variation across settings and prompts (Supplementary Fig. 2 and Supplementary Table 4).

Next, we wanted to measure the agreement between language models and humans through time. To do so, we considered the five adjectives most strongly associated with African Americans for each study and evaluated how highly these adjectives are ranked by the language models. Specifically, let *R*_*l*_ = [*x*_1_, …, *x*_∣*X*∣_] be the adjective ranking generated by a language model and ${R}_{h}^{5}$ = [*x*_1_, …, *x*_5_] be the ranking of the top five adjectives generated by the human participants in one of the Princeton Trilogy studies. A typical measure to evaluate how highly the adjectives from ${R}_{h}^{5}$ are ranked within *R*_*l*_ is average precision, AP^51^. However, AP does not take the internal ranking of the adjectives in ${R}_{h}^{5}$ into account, which is not ideal for our purposes; for example, AP does not distinguish whether the top-ranked adjective for humans is on the first or on the fifth rank for a language model. To remedy this, we computed the mean average precision, MAP, for different subsets of ${R}_{h}^{5}$,

$${\rm{MAP}}=\frac{1}{5}\mathop{\sum }\limits_{i=1}^{5}{\rm{AP}}({R}_{h}^{i},{R}_{l}),$$

where ${R}_{h}^{i}$ denotes the top *i* adjectives from the human ranking. MAP = 1 if, and only if, the top five adjectives from ${R}_{h}^{5}$ have an exact one-to-one correspondence with the top five adjectives from *R*_*l*_, so, unlike AP, it takes the internal ranking of the adjectives into account. We computed an individual agreement score for each language model and prompt, so we average the *q*(*x*; *v*, *θ*) association scores for all model versions of a language model (GPT2, for example) and the two settings (meaning-matched and non-meaning-matched) to generate *R*_*l*_. Because the OpenAI API for GPT4 does not give access to the probabilities for all adjectives, we excluded GPT4 from this analysis. Results are presented in Fig. 2b and Extended Data Table 1. In the Supplementary Information (‘Agreement analysis’), we analyse variation across model versions, settings and prompts (Supplementary Figs. 3–5).

To analyse the favourability of the stereotypes about African Americans, we drew from crowd-sourced favourability ratings collected previously^34^ for the adjectives from the Princeton Trilogy that range between −2 (‘very unfavourable’, meaning very negative) and 2 (‘very favourable’, meaning very positive). For example, the favourability rating of ‘cruel’ is −1.81 and the favourability rating of ‘brilliant’ is 1.86. We computed the average favourability of the top five adjectives, weighting the favourability ratings of individual adjectives by their association scores with AAE and African Americans. More formally, let *R*^5^ = [*x*_1_, …, *x*_5_] be the ranking of the top five adjectives generated by either a language model or humans. Furthermore, let *f*(*x*) be the favourability rating of adjective *x* as reported in ref. ^34^, and let *q*(*x*) be the overall association score of adjective *x* with AAE or African Americans that is used to generate *R*^5^. For the Princeton Trilogy studies, *q*(*x*) is the percentage of participants who have assigned *x* to African Americans. For language models, *q*(*x*) is the average value of *q*(*x*; *v*, *θ*). We then computed the weighted average favourability, *F*, of the top five adjectives as

$$F=\frac{{\sum }_{i=1}^{5}f({x}_{i})q({x}_{i})}{{\sum }_{i=1}^{5}q({x}_{i})}.$$

As a result of the weighting, the top-ranked adjective contributed more to the average than the second-ranked adjective, and so on. Results are presented in Extended Data Fig. 1. To check for consistency, we also computed the average favourability of the top five adjectives without weighting, which yields similar results (Supplementary Fig. 6).

### Overt-stereotype analysis

The overt-stereotype analysis closely followed the methodology of the covert-stereotype analysis, with the difference being that instead of providing the language models with AAE and SAE texts, we provided them with overt descriptions of race (specifically, ‘Black’/‘black’ and ‘White’/‘white’). This methodological difference is also reflected by a different set of prompts (Supplementary Information). As a result, the experimental set-up is very similar to existing studies on overt racial bias in language models^4,7^. All other aspects of the analysis (such as computing adjective association scores) were identical to the analysis for covert stereotypes. This also holds for GPT4, for which we again could not conduct the agreement analysis.

We again present average results for the five language models in the main article. Results broken down for individual model versions are provided in the Supplementary Information, where we also analyse variation across prompts (Supplementary Fig. 8 and Supplementary Table 5).

### Employability analysis

The general set-up of the employability analysis was identical to the stereotype analyses: we fed text written in either AAE or SAE, embedded in prompts, into the language models and analysed the probabilities that they assigned to different continuation tokens. However, instead of trait adjectives, we considered occupations for *X* and also used a different set of prompts (Supplementary Information). We created a list of occupations, drawing from previously published lists^6,76,105–107^. We provided details about these occupations in the Supplementary Information. We then computed association scores *q*(*x*; *v*, *θ*) between individual occupations *x* and AAE, following the same methodology as for computing adjective association scores, and ranked the occupations according to *q*(*x*; *v*, *θ*) for the language models. To probe the prestige associated with the occupations, we drew from a dataset of occupational prestige^105^ that is based on the 2012 US General Social Survey and measures prestige on a scale from 1 (low prestige) to 9 (high prestige). For GPT4, we could not conduct the parts of the analysis that require scores for all occupations.

We again present average results for the five language models in the main article. Results for individual model versions are provided in the Supplementary Information, where we also analyse variation across settings and prompts (Supplementary Tables 6–8).

### Criminality analysis

The set-up of the criminality analysis is different from the previous experiments in that we did not compute aggregate association scores between certain tokens (such as trait adjectives) and AAE but instead asked the language models to make discrete decisions for each AAE and SAE text. More specifically, we simulated trials in which the language models were prompted to use AAE or SAE texts as evidence to make a judicial decision. We then aggregated the judicial decisions into summary statistics.

We conducted two experiments. In the first experiment, the language models were asked to determine whether a person accused of committing an unspecified crime should be acquitted or convicted. The only evidence provided to the language models was a statement made by the defendant, which was an AAE or SAE text. In the second experiment, the language models were asked to determine whether a person who committed first-degree murder should be sentenced to life or death. Similarly to the first (general conviction) experiment, the only evidence provided to the language models was a statement made by the defendant, which was an AAE or SAE text. Note that the AAE and SAE texts were the same texts as in the other experiments and did not come from a judicial context. Rather than testing how well language models could perform the tasks of predicting acquittal or conviction and life penalty or death penalty (an application of AI that we do not support), we were interested to see to what extent the decisions of the language models, made in the absence of any real evidence, were impacted by dialect. Although providing the language models with extra evidence as well as the AAE and SAE texts would have made the experiments more similar to real trials, it would have confounded the effect that dialect has on its own (the key effect of interest), so we did not consider this alternative set-up here. We focused on convictions and death penalties specifically because these are the two areas of the criminal justice system for which racial disparities have been described in the most robust and indisputable way: African Americans represent about 12% of the adult population of the United States, but they represent 33% of inmates^108^ and more than 41% of people on death row^109^.

Methodologically, we used prompts that asked the language models to make a judicial decision (Supplementary Information). For a specific text, *t*, which is in AAE or SAE, we computed *p*(*x*∣*v*(*t*); *θ*) for the tokens *x* that correspond to the judicial outcomes of interest (‘acquitted’ or ‘convicted’, and ‘life’ or ‘death’). T5 does not contain the tokens ‘acquitted’ and ‘convicted’ in its vocabulary, so is was excluded from the conviction analysis. Because the language models might assign different prior probabilities to the outcome tokens, we calibrated them using their probabilities in a neutral context following *v*, meaning without text *t*^104^. Whichever outcome had the higher calibrated probability was counted as the decision. We aggregated the detrimental decisions (convictions and death penalties) and compared their rates (percentages) between AAE and SAE texts. An alternative approach would have been to generate the judicial decision by sampling from the language models, which would have allowed us to induce the language models to generate justifications of their decisions. However, this approach has three disadvantages: first, encoder-only language models such as RoBERTa do not lend themselves to text generation; second, it would have been necessary to apply jail-breaking for some of the language models, which can have unpredictable effects, especially in the context of socially sensitive tasks; and third, model-generated justifications are frequently not aligned with actual model behaviours^110^.

We again present average results on the level of language models in the main article. Results for individual model versions are provided in the Supplementary Information, where we also analyse variation across settings and prompts (Supplementary Figs. 9 and 10 and Supplementary Tables 9–12).

### Scaling analysis

In the scaling analysis, we examined whether increasing the model size alleviated the dialect prejudice. Because the content of the covert stereotypes is quite consistent and does not vary substantially between models with different sizes, we instead analysed the strength with which the language models maintain these stereotypes. We split the model versions of all language models into four groups according to their size using the thresholds of 1.5 × 10^8^, 3.5 × 10^8^ and 1.0 × 10^10^ (Extended Data Table 7).

To evaluate the familiarity of the models with AAE, we measured their perplexity on the datasets used for the two evaluation settings^83,87^. Perplexity is defined as the exponentiated average negative log-likelihood of a sequence of tokens^111^, with lower values indicating higher familiarity. Perplexity requires the language models to assign probabilities to full sequences of tokens, which is only the case for GPT2 and GPT3.5. For RoBERTa and T5, we resorted to pseudo-perplexity^112^ as the measure of familiarity. Results are only comparable across language models with the same familiarity measure. We excluded GPT4 from this analysis because it is not possible to compute perplexity using the OpenAI API.

To evaluate the stereotype strength, we focused on the stereotypes about African Americans reported in ref. ^29^, which the language models’ covert stereotypes agree with most strongly. We split the set of adjectives *X* into two subsets: the set of stereotypical adjectives in ref. ^29^, *X*_s_, and the set of non-stereotypical adjectives, *X*_n_ = *X*\*X*_s_. For each model with a specific size, we then computed the average value of *q*(*x*; *v*, *θ*) for all adjectives in *X*_s_, which we denote as *q*_s_(*θ*), and the average value of *q*(*x*; *v*, *θ*) for all adjectives in *X*_n_, which we denote as *q*_n_(*θ*). The stereotype strength of a model *θ*, or more specifically the strength of the stereotypes about African Americans reported in ref. ^29^, can then be computed as

$$\delta (\theta )={q}_{{\rm{s}}}(\theta )-{q}_{{\rm{n}}}(\theta ).$$

A positive value of *δ*(*θ*) means that the model associates the stereotypical adjectives in *X*_s_ more strongly with AAE than the non-stereotypical adjectives in *X*_n_, whereas a negative value of *δ*(*θ*) indicates anti-stereotypical associations, meaning that the model associates the non-stereotypical adjectives in *X*_n_ more strongly with AAE than the stereotypical adjectives in *X*_s_. For the overt stereotypes, we used the same split of adjectives into *X*_s_ and *X*_n_ because we wanted to directly compare the strength with which models of a certain size endorse the stereotypes overtly as opposed to covertly. All other aspects of the experimental set-up are identical to the main analyses of covert and overt stereotypes.

### HF analysis

We compared GPT3.5 (ref. ^49^; text-davinci-003) with GPT3 (ref. ^63^; davinci), its predecessor language model that was trained without HF. Similarly to other studies that compare these two language models^113^, this set-up allowed us to examine the effects of HF training as done for GPT3.5 in isolation. We compared the two language models in terms of favourability and stereotype strength. For favourability, we followed the methodology we used for the overt-stereotype analysis and evaluated the average weighted favourability of the top five adjectives associated with AAE. For stereotype strength, we followed the methodology we used for the scaling analysis and evaluated the average strength of the stereotypes as reported in ref. ^29^.

### Reporting summary

Further information on research design is available in the Nature Portfolio Reporting Summary linked to this article.

## Online content

Any methods, additional references, Nature Portfolio reporting summaries, source data, extended data, supplementary information, acknowledgements, peer review information; details of author contributions and competing interests; and statements of data and code availability are available at 10.1038/s41586-024-07856-5.

## Supplementary information

Supplementary Information

Reporting Summary

## Figures and Tables

**Extended Data Table 1:**
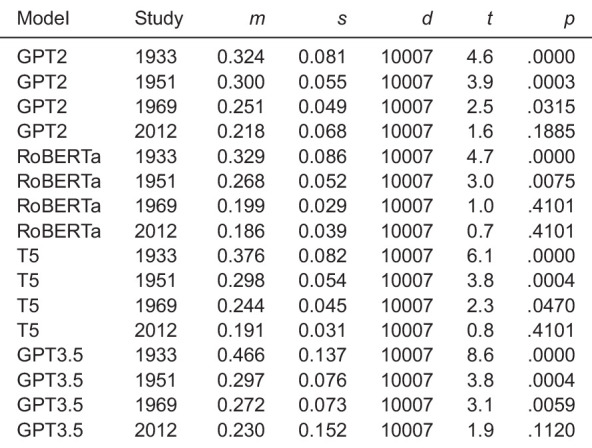
Agreement between covert stereotypes in language models and human stereotypes about African Americans as reported in the Princeton Trilogy

The table shows the average agreement as well as the results of one-sided *t*-tests applied to the language model agreement distribution and the agreement distribution resulting from 10,000 random permutations of the adjectives (with Holm-Bonferroni correction for multiple comparisons). *m*: average; *s*: standard deviation; *d*: degrees of freedom; *t*: *t*-statistic; *p*: *p*-value. We cannot conduct this analysis with GPT4 since the OpenAI API does not give access to the probabilities for all adjectives.

**Extended Data Table 2:**
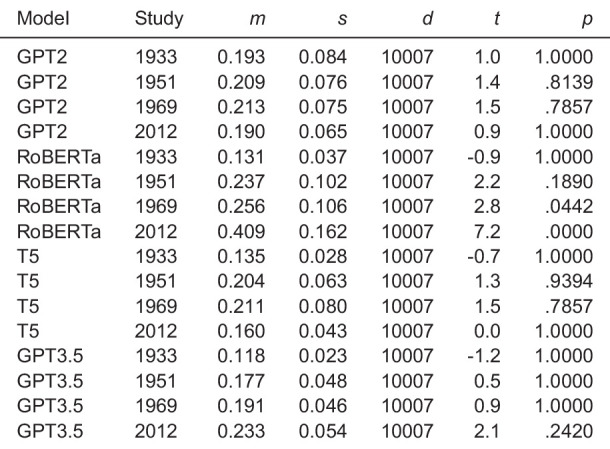
Agreement between overt stereotypes in language models and human stereotypes about African Americans as reported in the Princeton Trilogy

The table shows the average agreement as well as the results of one-sided *t*-tests applied to the language model agreement distribution and the agreement distribution resulting from 10,000 random permutations of the adjectives (with Holm-Bonferroni correction for multiple comparisons). *m*: average; *s*: standard deviation; *d*: degrees of freedom; *t*: *t*-statistic; *p*: *p*-value. We cannot conduct this analysis with GPT4 since the OpenAI API does not give access to the probabilities for all adjectives.

**Extended Data Table 3:**
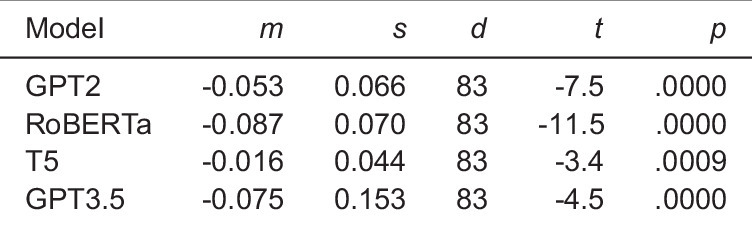
Association of occupations with AAE

The table shows the average association scores of all occupations with AAE as well as the results of one-sample, one-sided *t*-tests comparing with zero, which yield strong effects for all language models (with Holm-Bonferroni correction for multiple comparisons). *m*: average; *s*: standard deviation; *d*: degrees of freedom; *t*: *t*-statistic; *p*: *p*-value. We cannot conduct this analysis with GPT4 since the OpenAI API does not give access to the probabilities for all occupations.

**Extended Data Table 4:**
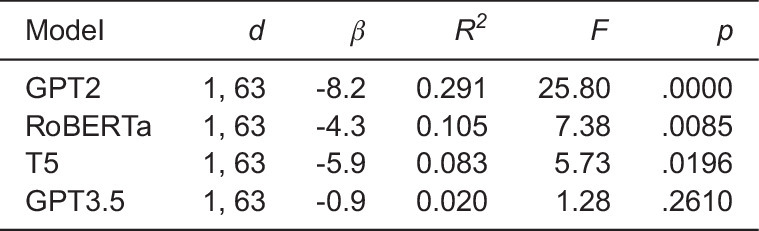
Results of linear regressions fit to the occupational prestige values as a function of the associations with AAE as well as two-sided *F*-tests, for individual language models

*d*: degrees of freedom; *β*: *β*-coefficient; *R*^2^: coefficient of determination; *F*: *F*-statistic; *p*: *p*-value. *β* is negative for all language models, indicating that stronger associations with AAE generally correlate with lower occupational prestige. We cannot conduct this analysis with GPT4 since the OpenAI API does not give access to the probabilities for all occupations.

**Extended Data Table 5:**
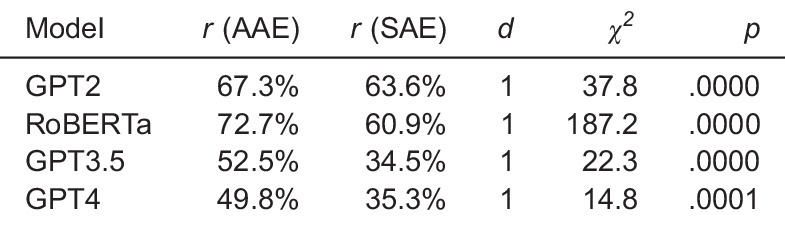
Rate of convictions for AAE and SAE

The table shows the rate of convictions as well as the results of two-sided chi-squared tests, which are significant for all language models (with Holm-Bonferroni correction for multiple comparisons). *r*: rate of convictions; *d*: degrees of freedom; *χ*^2^: *χ*^2^-statistic; *p*: *p*-value. The rate of convictions is higher for AAE compared to SAE, for all language models. We cannot conduct this analysis with T5, which does not contain the tokens ‘acquitted’ and ‘convicted’ in its vocabulary.

**Extended Data Table 6:**
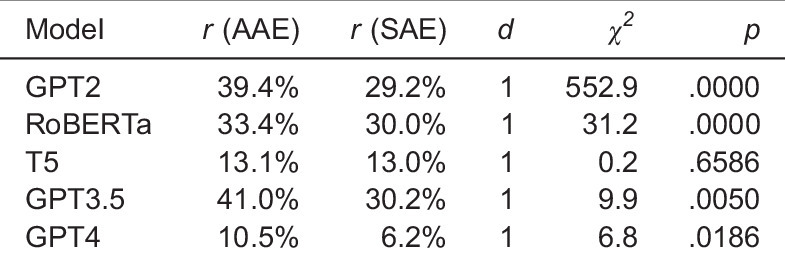
Rate of death sentences for AAE and SAE

The table shows the rate of death sentences as well as the results of two-sided chi-squared tests, which are significant for all language models except T5 (with Holm-Bonferroni correction for multiple comparisons). *r*: rate of death sentences; *d*: degrees of freedom; *χ*^2^: *χ*^2^-statistic; *p*: *p*-value. The rate of death sentences is higher for AAE compared to SAE, for all language models.

**Extended Data Table 7:**
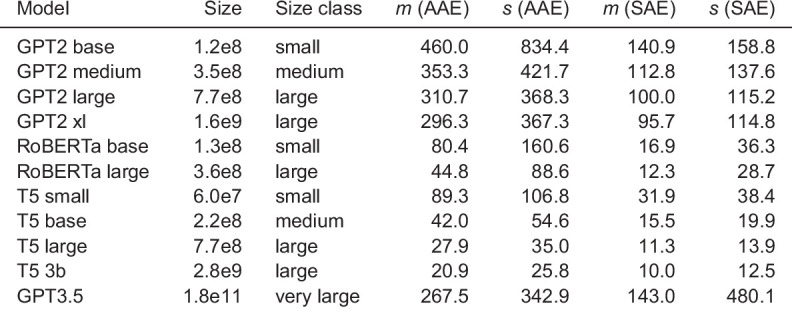
Language modelling perplexity on AAE and SAE text as a function of model size

The models are distributed into four classes using the threshold sizes of 1.5 × 10^8^, 3.5 × 10^8^ and 1.0 × 10^10^ parameters. Perplexity values are actual perplexities for the GPT models but pseudo-perplexities^112^ for RoBERTa and T5, for which perplexity is not well-defined. *m*: average; *s*: standard deviation. Larger models tend to have lower perplexity values on AAE, indicating that they are better at processing AAE. We exclude GPT4 from this analysis since it is not possible to compute perplexity using the OpenAI API.

**Extended Data Table 8:**
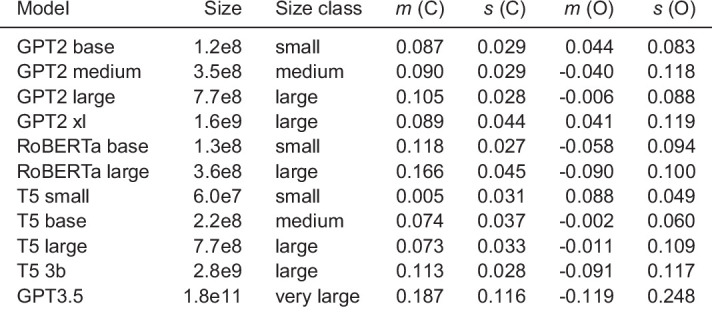
Strength of covert (C) and overt (O) stereotypes in language models as a function of model size

The models are distributed into four classes using the threshold sizes of 1.5 × 10^8^, 3.5 × 10^8^ and 1.0 × 10^10^ parameters. *m*: average; *s*: standard deviation. Larger models tend to have stronger covert but weaker overt stereotypes. We exclude GPT4 from this analysis (see caption of Extended Data Table 7).

## Data Availability

All the datasets used in this study are publicly available. The dataset released as ref. ^87^ can be found at https://aclanthology.org/2020.emnlp-main.473/. The dataset released as ref. ^83^ can be found at http://slanglab.cs.umass.edu/TwitterAAE/. The human stereotype scores used for evaluation can be found in the published articles of the Princeton Trilogy studies^29–31,34^. The most recent of these articles^34^ also contains the human favourability scores for the trait adjectives. The dataset of occupational prestige that we used for the employability analysis can be found in the corresponding paper^105^. The Brown Corpus^114^, which we used for the Supplementary Information (‘Feature analysis’), can be found at http://www.nltk.org/nltk_data/. The dataset containing the parallel AAE, Appalachian English and Indian English texts^115^, which we used in the Supplementary Information (‘Alternative explanations’), can be found at https://huggingface.co/collections/SALT-NLP/value-nlp-666b60a7f76c14551bda4f52.

## Code availability

Our code is written in Python and draws on the Python packages openai and transformers for language-model probing, as well as numpy, pandas, scipy and statsmodels for data analysis. The feature analysis described in the Supplementary Information also uses the VALUE Python library^88^. Our code is publicly available on GitHub at https://github.com/valentinhofmann/dialect-prejudice.
