# Supplementary material for: AI generates covertly racist decisions about people based on their dialect
Source: Nature. 2024 Aug 28;633(8028):147–54. doi: 10.1038/s41586-024-07856-5 (PMC11374696; doi:10.1038/s41586-024-07856-5)
Supplement: Supplementary file 2 — Reporting Summary [file 41586_2024_7856_MOESM2_ESM.pdf]

nature portfolio

Corresponding author(s): Valentin Hofmann, Sharese King

Last updated by author(s): Jun 21, 2024

Reporting Summary

Nature Portfolio wishes to improve the reproducibility of the work that we publish. This form provides structure for consistency and transparency in reporting. For further information on Nature Portfolio policies, see our Editorial Policies and the Editorial Policy Checklist.

Statistics

For all statistical analyses, confirm that the following items are present in the figure legend, table legend, main text, or Methods section.

n/a	Confirmed	
☐	☒	The exact sample size (*n*) for each experimental group/condition, given as a discrete number and unit of measurement
☐	☒	A statement on whether measurements were taken from distinct samples or whether the same sample was measured repeatedly
☐	☒	The statistical test(s) used AND whether they are one- or two-sided<br>*Only common tests should be described solely by name; describe more complex techniques in the Methods section.*
☐	☒	A description of all covariates tested
☐	☒	A description of any assumptions or corrections, such as tests of normality and adjustment for multiple comparisons
☐	☒	A full description of the statistical parameters including central tendency (e.g. means) or other basic estimates (e.g. regression coefficient) AND variation (e.g. standard deviation) or associated estimates of uncertainty (e.g. confidence intervals)
☐	☒	For null hypothesis testing, the test statistic (e.g. *F*, *t*, *r*) with confidence intervals, effect sizes, degrees of freedom and *P* value noted<br>*Give P values as exact values whenever suitable.*
☒	☐	For Bayesian analysis, information on the choice of priors and Markov chain Monte Carlo settings
☐	☒	For hierarchical and complex designs, identification of the appropriate level for tests and full reporting of outcomes
☐	☒	Estimates of effect sizes (e.g. Cohen's *d*, Pearson's *r*), indicating how they were calculated

Our web collection on statistics for biologists contains articles on many of the points above.

Software and code

Policy information about availability of computer code

Data collection	We used Python 3.10 to probe the language models. Specifically, we drew upon the package openai 0.28.1 to probe GPT3.5 and GPT4, and transformers 4.36.2 to probe GPT2, RoBERTa, and T5.
Data analysis	Data analysis was performed in Python 3.10. The specific packages we used were numpy 1.22.4, pandas 1.5.2, scipy 1.7.3, and statsmodels 0.13.2. All code used for data analysis can be found at https://github.com/valentinhofmann/dialect-prejudice.

For manuscripts utilizing custom algorithms or software that are central to the research but not yet described in published literature, software must be made available to editors and reviewers. We strongly encourage code deposition in a community repository (e.g. GitHub). See the Nature Portfolio guidelines for submitting code & software for further information.

Data

Policy information about availability of data

All manuscripts must include a data availability statement. This statement should provide the following information, where applicable:

- Accession codes, unique identifiers, or web links for publicly available datasets
- A description of any restrictions on data availability
- For clinical datasets or third party data, please ensure that the statement adheres to our policy

All datasets used in this study are publicly available. The dataset released by Groenwold et al. (2020) can be found at https://aclanthology.org/2020.emnlp-main.473/. The dataset released by Blodgett et al. (2016) can be found at http://slanglab.cs.umass.edu/TwitterAAE/. The human stereotype scores used for

evaluation can be found in the published articles of the Princeton Trilogy studies (Katz and Braly, 1933; Gilbert, 1951; Karlins et al., 1969; Bergsieker et al., 2012). The most recent of these articles (Bergsieker et al., 2012) also contains the human favorability scores for the trait adjectives. The dataset of occupational prestige that we use in the employability analysis can be found in the corresponding paper (Smith and Son, 2014). The Brown Corpus (Francis and Kucera, 1979), which is used in the Supplementary Information (Feature analysis), can be found at http://www.nltk.org/nltk data/. The dataset containing the parallel African American English, Appalachian English, and Indian English texts (Ziems et al., 2023), which is used in the Supplementary Information (Alternative explanations), can be found at https://huggingface.co/collections/SALT-NLP/value-nlp-666b60a7f76c14551bda4f52.

Research involving human participants, their data, or biological material

Policy information about studies with human participants or human data. See also policy information about sex, gender (identity/presentation), and sexual orientation and race, ethnicity and racism.

Reporting on sex and gender	The study did not involve human participants.
Reporting on race, ethnicity, or other socially relevant groupings	The study did not involve human participants.
Population characteristics	The study did not involve human participants.
Recruitment	The study did not involve human participants.
Ethics oversight	The study did not involve human participants.

Note that full information on the approval of the study protocol must also be provided in the manuscript.

Field-specific reporting

Please select the one below that is the best fit for your research. If you are not sure, read the appropriate sections before making your selection.

☐ Life sciences ☒ Behavioural & social sciences ☐ Ecological, evolutionary & environmental sciences

For a reference copy of the document with all sections, see nature.com/documents/nr-reporting-summary-flat.pdf

Behavioural & social sciences study design

All studies must disclose on these points even when the disclosure is negative.

Study description	We relied on existing datasets of African American English and Standard American English texts, which we embedded in prompts and fed into language models. We then analyzed the predictions of the language models for the two types of input, in both qualitative and quantitative ways.
Research sample	The study did not involve human participants. We used African American English and Standard American English texts from publicly available datasets, specifically Groenwold et al. (2020) and Blodgett et al. (2016), which are among the only large-scale datasets containing both African American English and Standard American English texts available today. While the two datasets cover the most stigmatized canonical features of African American English shared among Black speakers cross-regionally, neither of them is representative of the fine-grained regional variability of African American English.
Sampling strategy	For the smaller of the two datasets (Groenwold et al., 2020), we used all available texts. For the larger of the two datasets (Blodgett et al., 2016), we randomly sampled texts such that the resulting dataset had a similar size as the dataset from Groenwold et al. (2020). This was important in order to ensure comparability of results across datasets and to make conducting the experiments feasible for a larger number of language models.
Data collection	The texts from the two datasets were embedded in prompts asking for properties of the speakers who have uttered the texts. For each analysis, we selected several different prompts in order to be able to test for consistency. We then drew upon Python packages, specifically openai 0.28.1 and transformers 4.36.2, to feed the filled prompts into the language models and retrieve their predictions. We chose the examined language models to cover the full spectrum of language models in use today, in terms of architecture, size, and overall model cababilities.
Timing	Experiments involving GPT2, RoBERTa, T5, and GPT3.5 were conducted in April and May 2023. Experiments involving GPT4 were conducted in January 2024.
Data exclusions	No data were excluded from the analyses.
Non-participation	The study did not involve human participants.
Randomization	The study did not involve human participants.

Reporting for specific materials, systems and methods

We require information from authors about some types of materials, experimental systems and methods used in many studies. Here, indicate whether each material, system or method listed is relevant to your study. If you are not sure if a list item applies to your research, read the appropriate section before selecting a response.

Materials & experimental systems

n/a	Involved in the study
☒	☐ Antibodies
☒	☐ Eukaryotic cell lines
☒	☐ Palaeontology and archaeology
☒	☐ Animals and other organisms
☒	☐ Clinical data
☒	☐ Dual use research of concern
☒	☐ Plants

Methods

n/a	Involved in the study
☒	☐ ChIP-seq
☒	☐ Flow cytometry
☒	☐ MRI-based neuroimaging

Plants

Seed stocks	*Report on the source of all seed stocks or other plant material used. If applicable, state the seed stock centre and catalogue number. If plant specimens were collected from the field, describe the collection location, date and sampling procedures.*
Novel plant genotypes	*Describe the methods by which all novel plant genotypes were produced. This includes those generated by transgenic approaches, gene editing, chemical/radiation-based mutagenesis and hybridization. For transgenic lines, describe the transformation method, the number of independent lines analyzed and the generation upon which experiments were performed. For gene-edited lines, describe the editor used, the endogenous sequence targeted for editing, the targeting guide RNA sequence (if applicable) and how the editor was applied.*
Authentication	*Describe any authentication procedures for each seed stock used or novel genotype generated. Describe any experiments used to assess the effect of a mutation and, where applicable, how potential secondary effects (e.g. second site T-DNA insertions, mosiacism, off-target gene editing) were examined.*
